# The effect of probe geometry on rind puncture resistance testing of maize stalks

**DOI:** 10.1186/s13007-020-00610-8

**Published:** 2020-05-08

**Authors:** Douglas D. Cook, Kyler Meehan, Levan Asatiani, Daniel J. Robertson

**Affiliations:** 1 Department of Mechanical Engineering, Brigham Young University, Provo, UT 84602 USA; 2 Division of Engineering, New York University–Abu Dhabi, Saadiyat Island, Abu Dhabi, United Arab Emirates; 3 Department of Mechanical Engineering, University of Idaho, 875 Perimeter Dr., MS 0902, Moscow, ID 83844 USA

**Keywords:** Maize, Stalk, Strength, Lodging, Rind, Penetration, Puncture

## Abstract

**Background:**

Stalk lodging (breaking of plant stems prior to harvest) is a major impediment to increasing agricultural yields of grain crops. Rind puncture resistance is commonly used to predict the lodging resistance of several crop species. However, there exist no standard operating procedures or suggested protocols for conducting rind penetration experiments. In addition, experimental details of rind penetration tests such as the shape and size of the penetrating probe are rarely reported in the literature. This has prevented meta-analysis of results and has likewise prevented key findings of past studies from being replicated. As a first step towards establishing an agreed upon measurement standard for rind puncture resistance this study investigates the effect of the puncturing probe’s geometry on test results.

**Results:**

Results demonstrate that probe geometry has a significant impact on test results. In particular, results showed that a 2 mm diameter chamfered probe produced stronger correlations with stalk bending strength than a 1.5 mm diameter pointed probe. The chamfered probe was also more strongly correlated with geometric features of the stalk that are known to influence stalk lodging resistance (e.g., rind thickness, diameter and section modulus). In addition, several alternative rind penetration metrics were investigated, and some were found to be superior to the most common rind penetration metric of maximum load.

**Conclusions:**

There is a need in the agricultural and plant science community to create agreed-upon operating procedures and testing standards related to mechanical traits of plant stems. In particular, a standardized probe geometry and insertion rate for rind penetration studies are needed to enable greater interoperability and meta-analysis of results. Probe shape and size should be reported in any study conducting rind penetration tests as these factors significantly impact test results.

## Background

Maize (*Zea mays*) is the most widely grown crop in the world [1], however stalk lodging (breaking of plant stalks prior to harvest) reduces global maize yields by approximately 5% annually [1–4]. Stalk lodging is a complex trait attributable to several anatomical, biochemical and physiological factors and it impedes production of several important crops [e.g., maize, rice (*Oryza sativa*), wheat (triticum), barley (*Hordeum vulgare*), sorghum (sorghum bicolor), sugarcane (*Saccharum officinarum*)]. The most commonly employed breeding metric for improving stalk lodging resistance simply relies on counting the number of lodged stalks prior to harvest (i.e.,  % lodged plants per plot). This approach is highly confounded by numerous factors, some of which include, location, weather and disease [2, 3, 5–8]. Other approaches to quantifying stalk lodging resistance have included measuring stalk crushing strength, dry weight, rind thickness and stalk bending strength, among others [5, 9–12]. An alternative method for predicting lodging susceptibility and stalk strength is rind penetration resistance. Several key advantages of rind penetration resistance are its non-destructive nature, ease of use, and low cost (i.e., it does not require overly expensive equipment, killing the plant, or conducting any specialized laboratory tests).

The rind penetration methodology involves forcing a small probe through a plant stalk or stem and measuring the maximum force required to penetrate the rind. This method has been used throughout most of the 20th century to investigate stalk strength and dates back to at least 1935 [13]. Several more recent studies have investigated the genetic architecture of rind penetration resistance and its relationship with morphological traits of stalks and stems [3, 14–18]. Even though numerous studies have utilized the rind penetration method there is no common protocol for preforming this measurement. As a result, the methodology varies from study to study making it difficult or impossible to directly compare results between studies. In addition, while it is generally understood in the broader literature that penetration measurements are dependent upon the shape and size of the impending probe there is no information or discussion of rind penetration probe shape and size in the maize literature. It is therefore unknown which probe geometry may provide the most reliable results for assessing stalk strength. The absence of documented operating procedures and testing standards for rind penetration measurements and the related inability of current researchers to reproduce key findings of past studies is likely why the methodology has not been widely employed by breeding programs.

The purpose of this study was to examine the mechanisms that affect rind penetration measurements of maize stalks thereby enabling a clearer understanding of the physical underpinnings of this measurement technique. In particular, we investigated the effect of probe geometry (shape and size), force application method (machine actuated vs. handheld), and puncture location (position along the stalk) on rind penetration measurements of maize stalks. We also examined several alternative rind penetration metrics in addition to maximum force and determined correlations with geometric, material, and structural information of the tested maize stalk samples. The results are expected to inform future studies and begin to lay the groundwork for the development of measurement standards for rind penetration resistance.

## Methods

### Plant materials

Maize stalks were sampled from two replicates of five commercially available hybrids of dent corn (maize) seeded at five planting densities (119,000, 104,000, 89,000, 74,000, and 59,000 plants ha^−1^) in two locations in Iowa. A total of 1000 plants were sampled (10 plants per each hybrid-planting density-replicate-location combination). Plants were cut just above the ear and just above the ground immediately prior to harvest and were placed on forced air dryers to reduce stalk moisture to approximately 10–15% moisture by weight. Dry and mature stalks were utilized as this state mimics the natural state of stalks in the field just prior to harvest (which is when they are most susceptible to late season stalk lodging) and enables storage of stalk samples without degradation.

### Measuring stalk geometry

All stalks were imaged using an X-ray computed tomography (CT) scanner (NorthStar Imaging, Rogers, MN) as described in [10]. The scan region of each stalk was centered on the most central node of the stalk sample. The scans allowed for accurate determination of rind thickness, cross-sectional diameter, and tissue density as described in [10]. The section modulus of each stalk was also determined. Section modulus is an engineering term used to describe the cross-sectional geometry of beams. In particular the section modulus is the geometric term that is used in engineering calculations to determine the flexural stiffness and flexural strength of beams in bending. It is calculated as:

1 $$\frac{{\pi (D d^{3} - \left( {D - 2 r} \right)\left( {d - 2 r)^{3} } \right)}}{32 d}$$

where *D* is the major diameter of the stalk, *d* is the minor diameter of the stalk and *r* is rind thickness. In other words, the major and minor diameter and rind thickness of stalks are naturally expected to be correlated with stalk bending strength. However, the section modulus represents the most appropriate way in which to simultaneously account for all the nonlinear relationships between these geometric factors and stalk bending strength. The derivation of the equation for section modulus is based on governing physics and theory (i.e., first principles) and is not merely an empirical or phenomenological relationship.

### Measuring stalk bending strength

An Instron Universal Testing System (Instron 5965, Instron Corp., Norwood, MA, USA) was utilized to conduct three point bending tests of stalk samples as described in [11, 19]. Synchronized force and displacement data were captured by Instron software (Bluehill 3.0). The most central node of each stalk was loaded while the most basal and apical nodes of the stalk were supported. This methodology produces the same failure types and patterns observed in naturally lodged stalks [20] and is therefore one of the most accurate methods of quantifying stalk-lodging resistance (i.e., stalk bending strength) on a plant-by-plant basis. The stalks typically failed (broke) just apical of the loaded node (i.e., in the same region the CT-scan was conducted).

Young’s Modulus (E) of the rind tissue of each stalk was estimated using principles and approximations of engineering beam theory as described in [21]. In particular, the Young’s Modulus of the rind was defined as

2 $$E = \frac{{a^{2} b^{2} }}{3IL}\emptyset$$

where *L* is the distance between the left and right supports, *a* and *b* are the distances from the left and right anvils to the point of applied load, $\emptyset$ is the slope of the force–deflection curve, and *I* is the moment of inertia of the rind determined from the CT scans as described in [5, 10]. This method ignores the contribution of the pith, which has been shown to be negligible [9, 21]. Derivation of Eq. 2 is given in [21, 22].

### Rind penetration measurements

Rind penetration measurements were acquired using the same universal testing system described above. The universal testing system provided a constant insertion velocity and a constant insertion angle during rind penetration experiments. Stalk samples were secured to a horizontal plate and oriented such that the minor axis of the stalk cross-section was vertical. Probes were lowered at a rate of 30 mm/s and synchronized force displacement data was recorded by the Instron software (Bluehill 3.0) at a rate of 100 Hz.

Each stalk was also submitted to a single rind penetration test in which the puncture force was applied manually (by hand). The same single researcher conducted all the manual penetration tests to eliminate any errors associated with inter-user variability. These tests were conducted to determine if the method of force actuation (machine-controlled test vs a hand actuated test) had a substantial influence on test results.

### Probe geometry

**Fig. 1:**
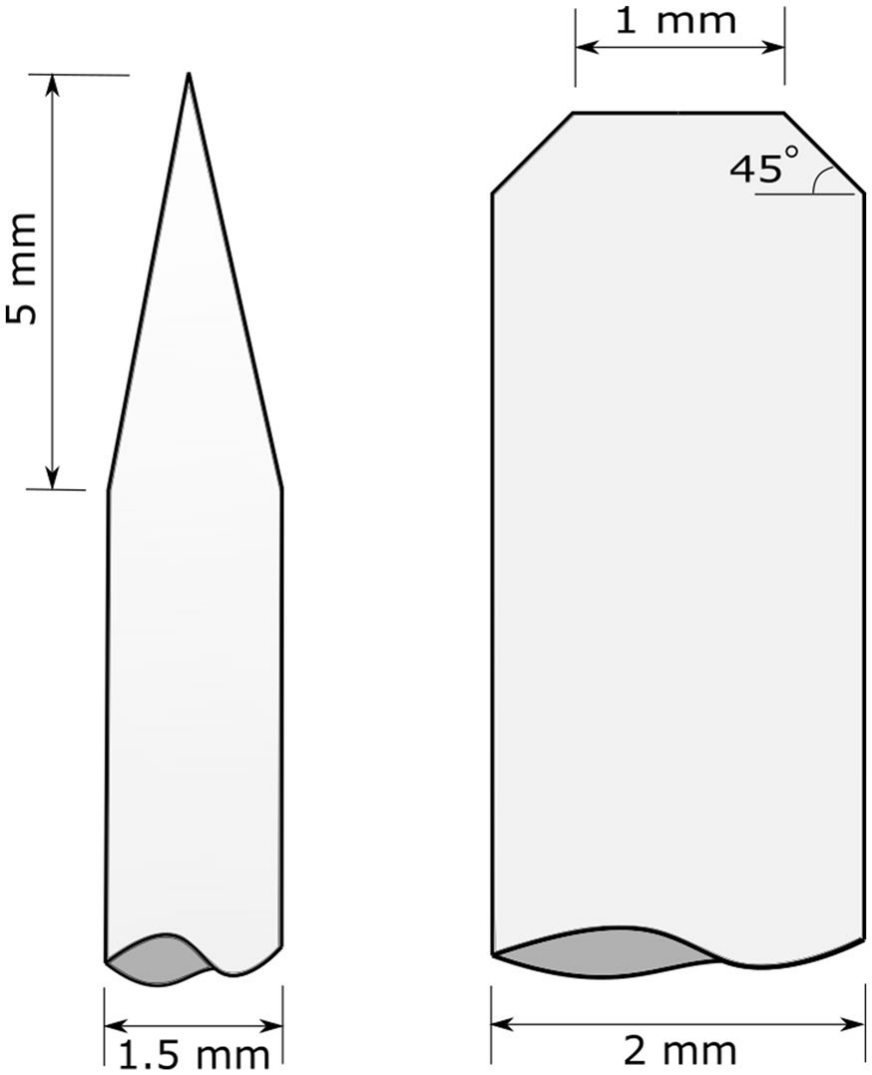
Rind penetration probe geometries utilized in the current study are shown. The pointed probe geometry is shown at left and the chamfered probe is shown at right

During preliminary testing, the authors investigated the use of over 15 unique rind penetration probes. Most of these were either too small (probes broke), inflicted significant damage to the stalk, or required forces greater than 200 N to puncture the stalk. These probes were eliminated, and two probe geometries were selected for further study. The first probe was 1.5 mm in diameter and tapered to a point over 5 mm (hereafter referred to as pointed probe). The second probe was 2 mm in diameter and had a 0.5 mm, 45° chamfer on its end (hereafter referred to as chamfered probe). Both probes were constructed of high-strength steel. The detailed geometry of each probe is displayed in Fig. 1.

### Puncture location

**Fig. 2:**
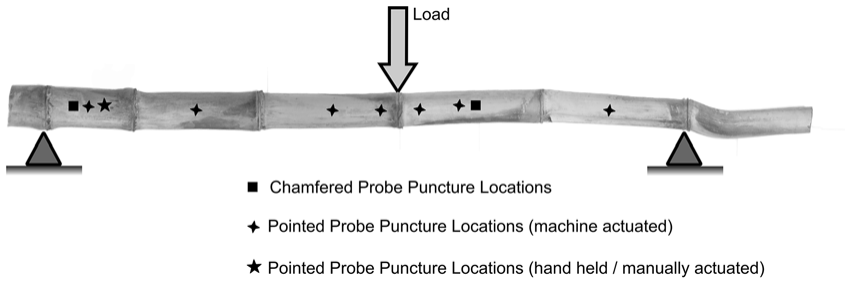
Image of typical maize stalk is shown with the locations of supports and applied load used during three-point-bending. The puncture locations of each rind penetration test are also depicted. The pointed probe was used to puncture the central portion of every internode as well as 5 cm apical and basal the node loaded in the three-point-bending test. The chamfered probe was used to puncture the central portion of the most basal internode and the central portion of the internode immediately apical of the applied three-point-bending load

Rind penetration tests were conducted at numerous locations on each stalk with each probe geometry. Figure 2 displays each probe geometry/puncture location combination. Chamfered probe penetration tests were conducted in two locations on each stalk. First, the central portion of the internode located immediately apical of the node loaded during the three-point-bending test (i.e., the most central internode) was punctured. Second, the chamfered probe was utilized to puncture each stalk in the center of the most basal internode. Pointed probe penetration tests were conducted in the central portion of each internode of each stalk as well as 5 cm apical and basal of the node loaded in the three-point-bending test. In addition, the most basal node of each stalk was submitted to an additional puncture test in which the pointed probe was forced through the stalk by hand (as opposed to by the Instron universal testing system). During all tests the probe was lowered until it had punctured the rind and entered the pith tissue. All rind puncture testing was conducted after flexural testing was complete.

The sampling strategy outlined in Fig. 2 (i.e., the choice of puncture locations) is fairly complex. In particular, some internodes were punctured by both chamfered and pointed probes whereas other internodes were only punctured by the pointed probe. Puncture locations were chosen to ensure the results of each test were not confounded by any previous testing that could have compromised the stalk. The top internodes of the stalk are smaller in diameter and are more easily damaged during puncture testing. Thus, top internodes were only punctured once as the authors were concerned that a single test could compromise the structural integrity of the internode thereby affecting subsequent testing. Top internodes were punctured with the pointed probe as it inflicted less structural damage as compared to the chamfered probe and hand actuated tests. In like manner the pointed probe was used to puncture 5 cm above and below the most central internode as the pointed probe geometry is less damaging to the stalk as compared to the chamfered probe. The middle portion of the most central internode was tested by both the pointed and chamfered probe so that results could be compared to CT scan data. The most basal internode is the largest diameter internode and is therefore the ideal location at which to conduct multiple puncture tests. For this reason, the most central portion of the basal internode was tested three times (one hand actuated and two machine actuated tests).

### Rind penetration metrics

**Fig. 3:**
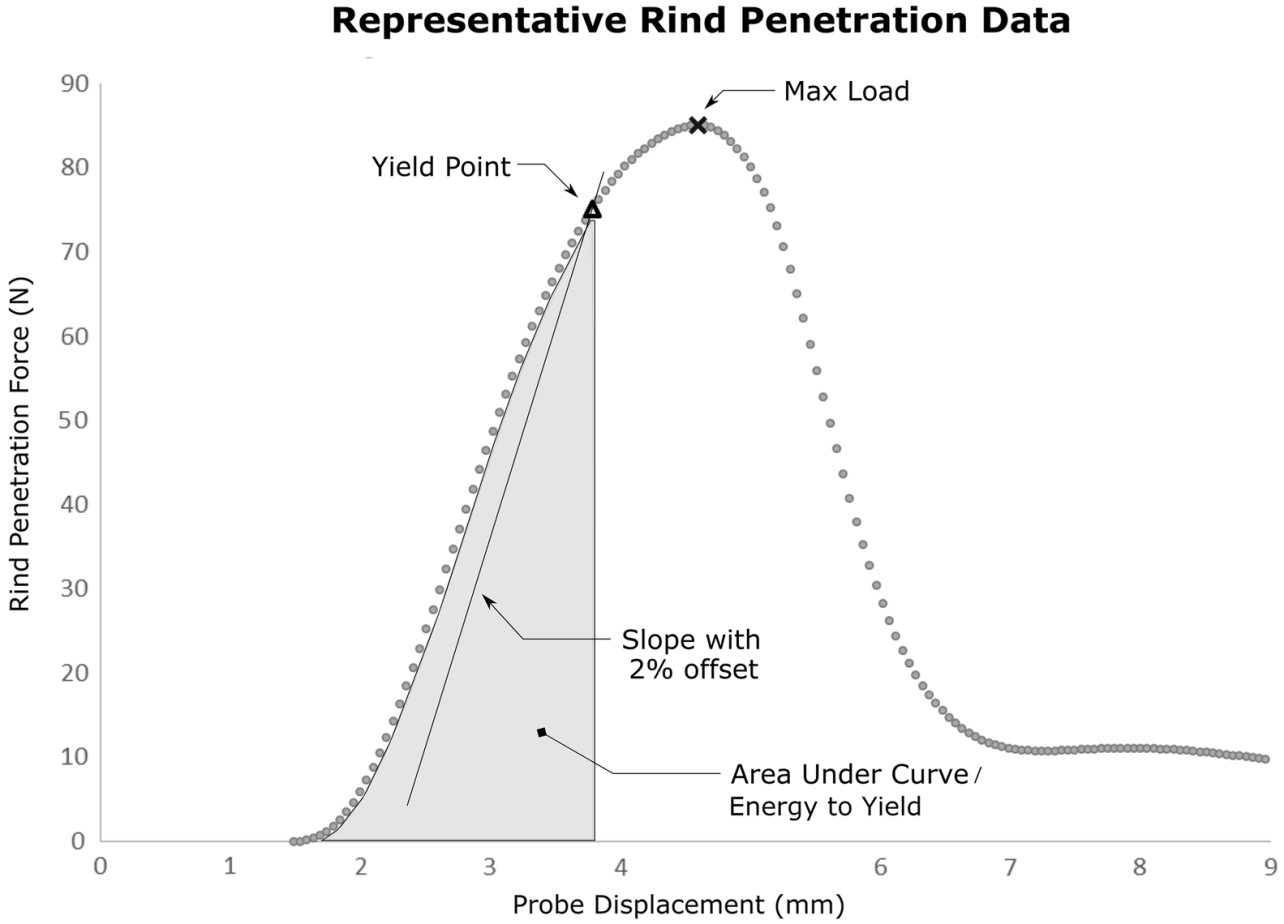
Representative data from a typical rind penetration experiment conducted using a universal testing system is depicted and several alternative rind penetration resistance measurements are demonstrated in addition to the typical measurement of maximum load. The slope of the linear portion of the curve is shown offset by 2% to illustrate the relationship between the slope and the yield point

The standard rind penetration protocol requires puncturing the rind of the stalk and measuring the maximum force encountered during the test. However, it is possible to compute several other metrics from rind penetration data. Figure 3 displays a typical force–displacement curve acquired from a single rind penetration experiment and highlights four alternative rind penetration computations. These alternative computations or metrics include the structural yield point, the slope of the linear portion of the curve, and the area under the curve (i.e., energy) up to the structural yield point. The structural yield point was calculated by offsetting the slope line by 2% of the deflection at the max load. The intersection between the offset average slope line and the force deflection curve was then defined as the structural yield point (see Fig. 3). Note that the *structural* yield point calculation used in this study is slightly different than the standard *material* yield point definition used by structural engineers. In particular, engineers typically define a *material* yield point using a 0.2% strain offset and not a 2% of deflection at max load offset. More information about yield point calculations can be found in [23]. For every rind penetration test except the hand operated tests, the 3 alternative metrics listed above were calculated. Alternative metrics were not calculated for the hand operated test because no displacement data was recorded during the manually actuated tests.

### Summary of rind penetration experiments

Table 1 displays a summary of the rind penetration experiments employed in the study including puncture locations, probe geometries, and number of rind penetration metrics recorded for each test.

**Table 1:** Summary of Rind Penetration Experiments

Actuation method	Probe geometry	Locations		Metrics	
		#	Description	# per location	Description
Hand actuated	Pointed	1	Most basal internode	1	Max load
Machine actuated	Pointed	8	Every internode and 5 cm apical and basal of most central node	4	Max load, slope, energy, yield point
Machine actuated	Chamfered	2	Most basal internode, most central internode	4	Max load, slope, energy, yield point

## Results

The results are divided into two major subsections. The first section focuses on the relationships between parameters of rind penetration tests and stalk bending strength. The second section focuses on relationships between stalk morphology and puncture test data.

## Relationships between puncture metrics and bending strength

### Data types and dimensions

The information presented below is composed of multiple data types, including geometric data, strength data, rind penetration data, and tissue data. As described above, bending strength, tissue properties, and geometric features were measured for each stalk at a single location near the central internode. Thus, these data have a sample size of approximately 1000. Rind puncture tests consisted of 4 rind penetration metrics (Fig. 3), each of which were obtained at multiple locations along the stalk (Table 1). For example, there were approximately 1000 tests performed with the sharp probe at the center of the second internode, which resulted in 1000 data points for each of the 4 puncture metrics. Univariate correlation analysis was performed to examine the relationship between bending strength and each predictor. Each of the R^2^ values presented below are based upon approximately 1000 bending tests and 1000 predictor values.

Rind penetration metrics were positively correlated with stalk bending strength across all testing locations and using both probe types. Coefficient of determination (R^2^) values ranged from a low of 0.13 (pointed probe at the 4th internode), to a high of 0.60 (chamfered probe at the most central internode). In the following sections we examine differences in the distributions of coefficient of determination values (R^2^) corresponding to different test metrics, probe geometries, and puncture locations.

### Probe geometry

**Fig. 4:**
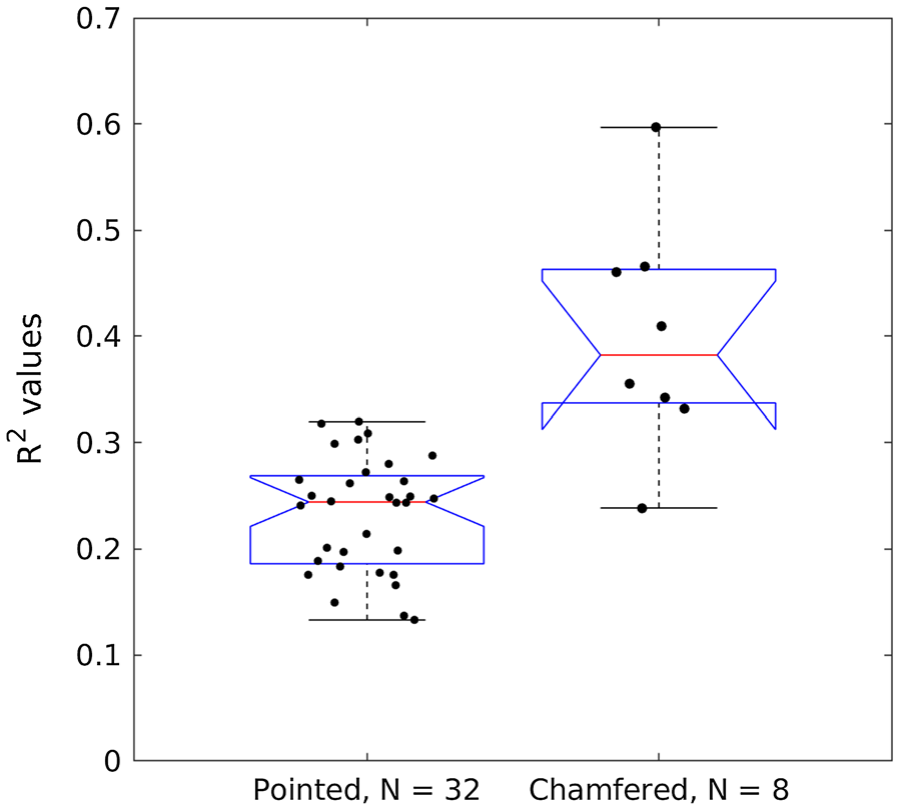
Box plots of R^2^ values for bending strength vs rind penetration resistance categorized by probe geometry. The central line of each box plot indicates the median value. The bottom and top edges of the box indicate 25th and 75th percentiles. Whiskers extend to the most extreme data point. Notches indicate statistical significance (i.e., 95% confidence interval of the median). Notches on the right hand box plot exceed the 25th percentile because of the relatively small sample size

A total of 32 R^2^ values were calculated for the pointed probe (4 metrics × 8 puncture locations) whereas 8 R^2^ values were calculated for the chamfered probe (4 metrics × 2 puncture locations). The chamfered probe was more highly correlated with bending strength than the pointed probe. In particular, the 32 R^2^ values from the pointed probe tests were statistically different than the 8 R^2^ values from the chamfered probe tests (two-sided t-test, p-value < 0.001). The mean R^2^ value for each probe geometry across all metrics and puncture locations are shown in Fig. 4, along with 95% confidence intervals. Examination of the distributions of these data revealed that the 75th percentile of the pointed probe R^2^ values were lower than the 25th percentile of the chamfered probe R^2^ values. The authors appreciate that numerous statistical analyses are available to test for differences between probe geometries. The statistical analysis presented above was chosen as it was believed to be most easily understood by a broad audience. The authors conducted several other statistical analyses to test for differences between probe geometries and reached the same conclusion each time. Namely that the chamfered probe is a better predictor of stalk strength as compared to the pointed probe (e.g., see Fig. 6).

### Rind penetration metrics

**Fig. 5:**
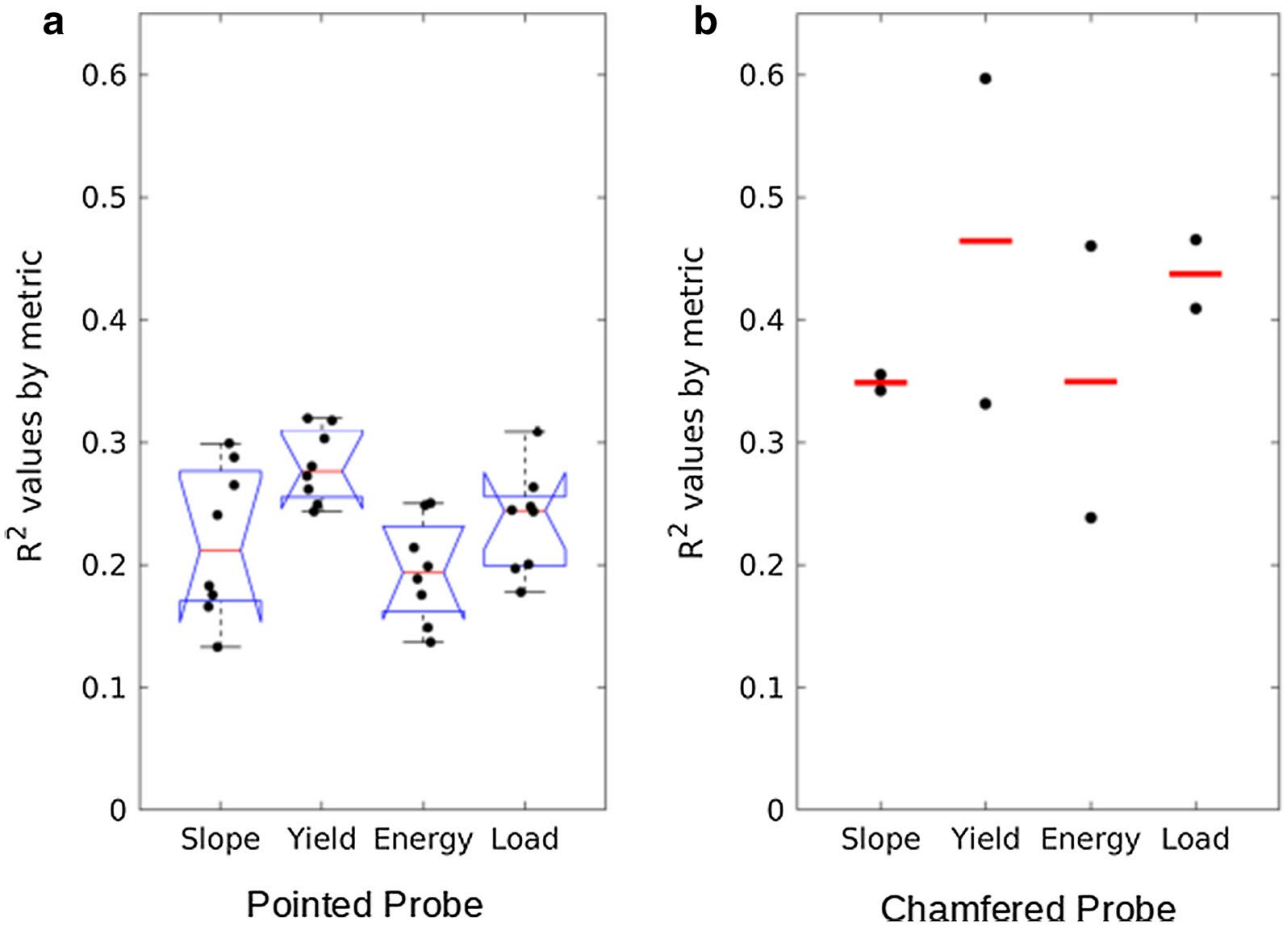
Distributions of R^2^ values between stalk strength and various rind penetration metrics. A: Boxplots of R^2^ values for the pointed probe (N = 8 in each group). The central line of each box plot indicates the median value. The bottom and top edges of the box indicate 25th and 75th percentiles. Whiskers extend to the most extreme data point. Notches indicate statistical significance (i.e., 95% confidence interval of the median). Note for some of the metrics the notches exceed the 25th percentile or 75th percentile because of the relatively small sample size. B: R^2^ values for the chamfered probe (N = 2 in each group). Horizontal lines indicate the mean value of the two R^2^ values

A total of 10 R^2^ values were calculated for the structural yield point, energy, slope, and max load metrics (8 machine actuated pointed probe tests + 2 machine actuated chamfered probe tests on each stalk). Across both probe types the yield point had the highest average correlation with bending strength, followed by maximum load. Figure 5 displays the R^2^ values of each rind penetration metric for both the pointed probe and the chamfered probe. Note bar charts are shown for the chamfered probe instead of box plots as the sample size was only N = 2 for each metric.

### Puncture location

**Fig. 6:**
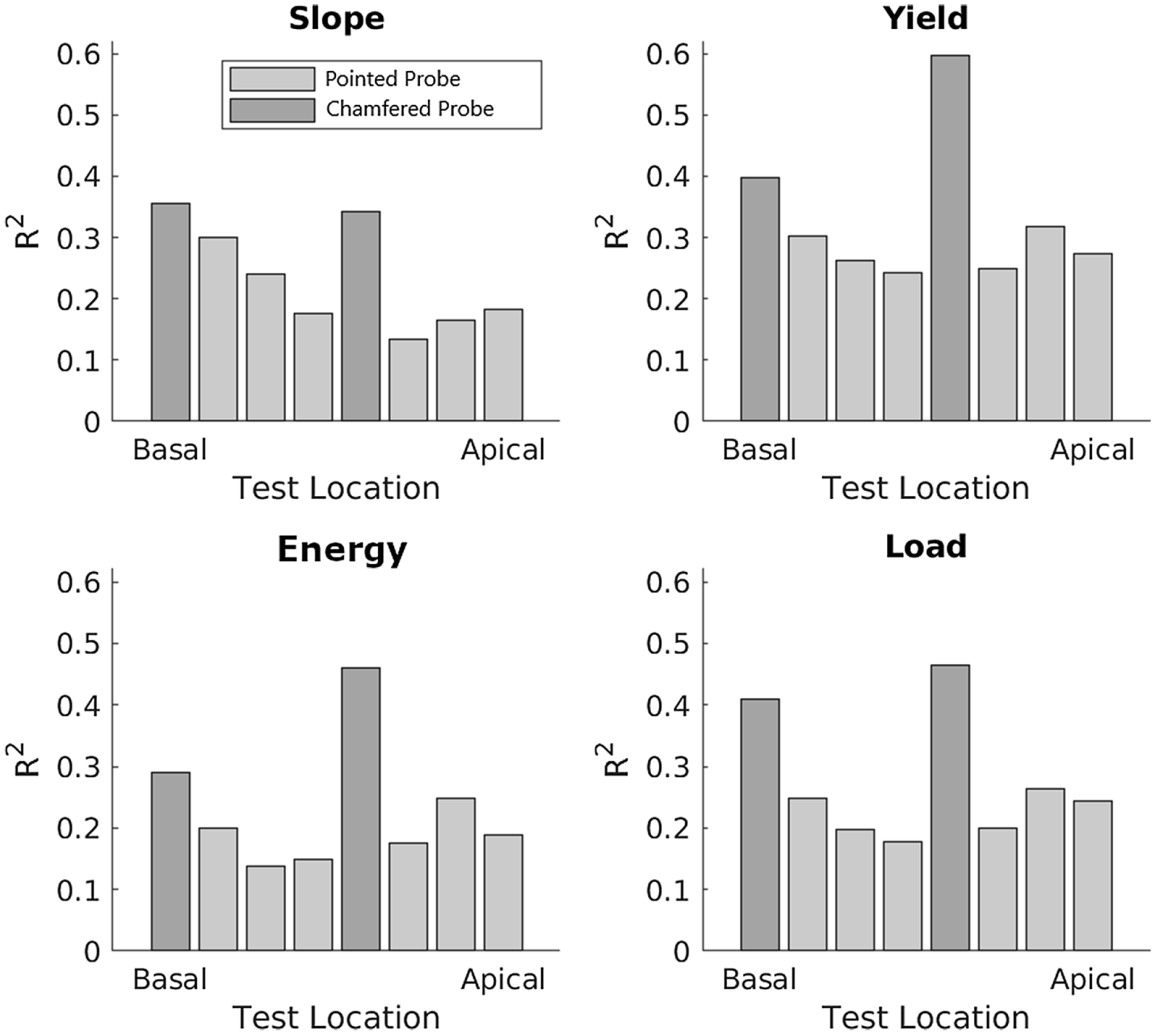
Correlations between rind puncture metrics and strength as a function of location along the stalk. Each bar chart represents a single metric. Whiskers are not present because each bar represents a single average R^2^ value. For each rind penetration metric the chamfered probe (dark grey bars) produced a stronger correlation with stalk bending strength than the pointed probe (light grey bars). When considering all rind penetration metrics the pointed probe did not demonstrate any consistent trends with regards to puncture location. However, the chamfered probe consistently produced higher correlations at the most central internode as opposed to the most basal internode across all rind penetration metrics

Individual R^2^ values for each rind penetration metric separated according to puncture location are presented in Fig. 6. The puncture locations 5 cm apical and basal of the center most node is not plotted as the correlations at these locations were less than at the center of the same internodes. This figure does not include confidence intervals for each bar because (in contrast to previous charts) each bar in Fig. 6 represents a single R^2^ value. The most noticeable feature of Fig. 6 is the contrast in R^2^ values between pointed and chamfered probes. The highest correlation between bending strength and rind puncture resistance was for the yield point metric when using the chamfered probe (Fig. 5). The next highest correlations were for energy and maximum load at the same test locations, with R^2^ values of 0.47 and 0.46, respectively. The chamfered probe tended to produce higher correlations when puncturing the most central internode of the stalk as opposed to the most basal internode of the stalk. In contrast, we were unable to confirm that the pointed probe exhibited any consistent pattern across puncture locations. For some of the metrics it appears that R^2^ values may decrease towards the center of the stalk, but we were unable to confirm this statistically (Fig. 6). For example, the highest correlation between the pointed probe slope metric and strength was at the base of the stalk. However, the highest correlations for the pointed probe yield point and energy metrics were near (but not at) the apical section of the stalk.

The purpose of Fig. 6 is to depict the lack of any consistent relationship between basal/apical location and any of the rind penetration metrics. While some patterns appear to be present for the pointed probe (light gray bars), the trends for the chamfered probe (dark gray bars) often indicate an opposite trend. The highest correlation was found between the yield point metric with the blunt probe at the central internode (Fig. 6).

### Hand tests vs. machine tests

While machine actuated tests were anticipated to be much more reliable than hand tests, this was not observed. Because the slope, yield point, and energy metrics require displacement data, only the maximum load metric can be extracted from tests performed by hand. Notwithstanding this limitation, the maximum load from hand tests had a similar R^2^ value as the machine actuated tests for maximum load. In particular, the hand actuated R^2^ values for max load was 0.26 whereas the average R^2^ value from machine actuated tests for max load were 0.28.

## Relationships between puncture measurements, stalk geometry and tissue properties

CT-images and three-point bending test were used to assess the relationship between rind penetration measurements (max load, slope, energy, and yield point) and stalk morphological and material properties (rind thickness, diameter, section modulus, bending strength, Young’s Modulus). Detailed geometric and tissue properties information were measured at the most central internode of each stalk sample (i.e., the internode immediately above the applied three-point-bending load). Both pointed probe and chamfered probe rind penetration tests were performed at this internode as well. Correlation analyses were performed on these data (from the central internode) to investigate how structural properties of the stalk (i.e., geometry and tissue properties) influence rind penetration metrics.

**Fig. 7:**
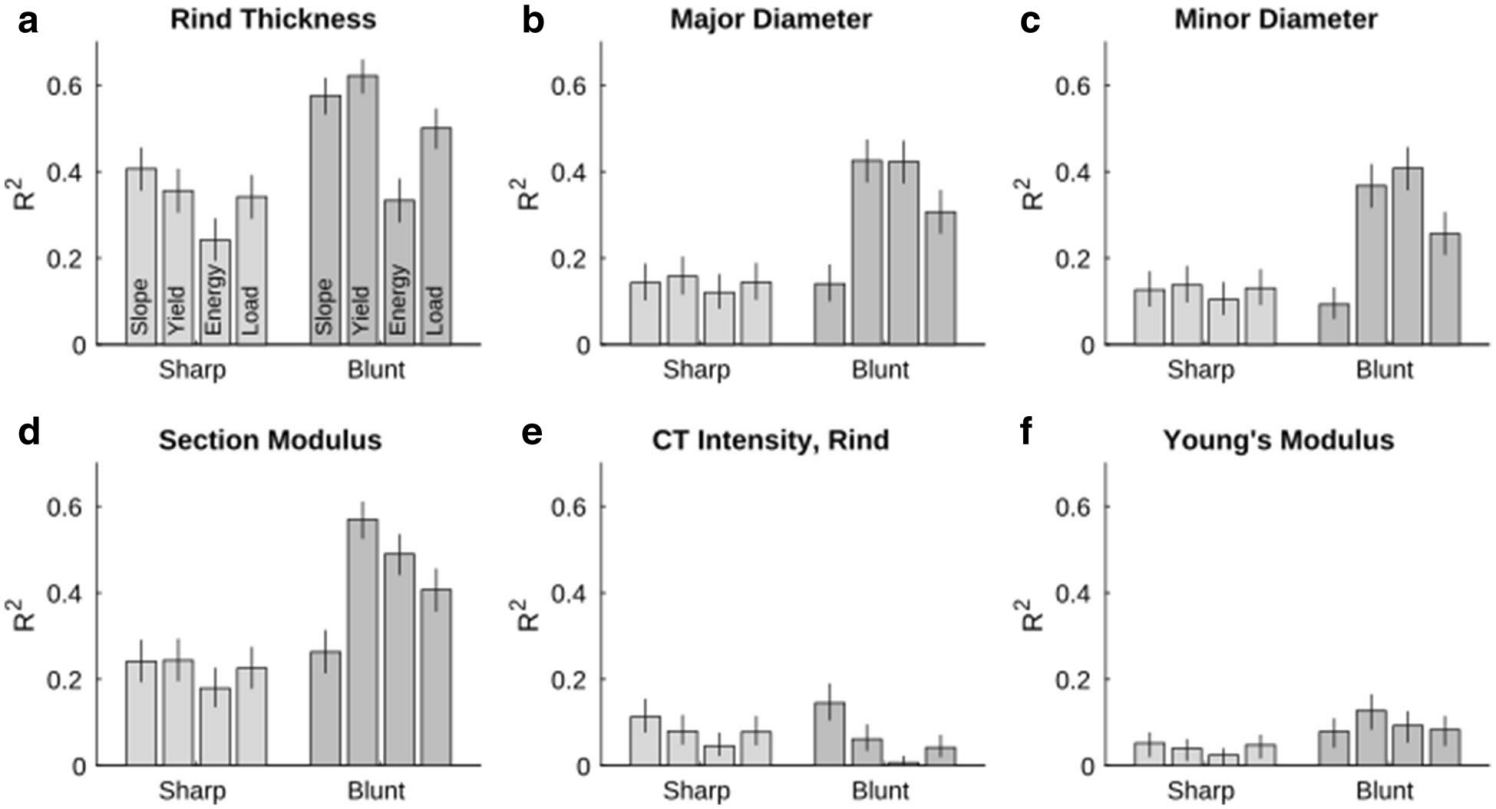
R^2^ values between various features of the maize stalk and rind penetration metrics. Whiskers represent 95% confidence intervals on each computed R^2^ value

Rind penetration metrics showed significant correlations with stalk geometry and stalk tissue properties. Rind penetration metrics were most highly correlated with rind thickness and section modulus, followed by stalk diameters. As shown in Fig. 7, the chamfered probe exhibited consistently higher correlations with stalk geometry than the pointed probe. Furthermore, various metrics exhibited varying levels of correlation with geometric parameters. The chamfered probe’s yield-point metric had the highest correlation with 4 stalk features and had the second-highest correlation for 1 additional feature (rind thickness, major diameter, section modulus, Young’s Modulus, and minor diameter, respectively).

Correlations between rind penetration metrics and tissue properties were statistically significant, but the correlation values were quite low. As seen in Fig. 7, the chamfered probe tended to have higher correlations not only with bending strength but with most geometric parameters as well. The correlations between CT intensity (i.e., the average CT intensity of the stalks cross-section which is related to tissue density) and rind puncture metrics demonstrated no statistical difference between the pointed and chamfered probes. An in depth analysis of the relationship between geometric features and stalk strength is presented in [10].

## Discussion

Results demonstrate that probe geometry (diameter and shape) has a statistically significant influence on the correlation between rind penetration tests and stalk bending strength. This is significant because most prior studies that have employed rind puncture tests have not provided information regarding the probe diameter or shape utilized in the study. The chamfered probe used in this study showed better correlations with stalk bending strength and morphological features of the stalk that are known to influence stalk strength as compared to the pointed probe.

### Rind puncture metrics

This study examined four different rind puncture metrics: slope, structural yield point, energy, and max load (see Fig. 3 for description of these metrics). The yield point metric was most strongly correlated to stalk bending strength. However, the max load metric was also moderately correlated to stalk bending strength and is easier to acquire as it only requires collection of force data but not displacement data. The yield point, slope, and energy metric require a second sensor channel to capture probe displacement throughout the progression of the puncture tests. Currently, there are no portable electronic systems to synchronously measure force and displacement of puncture test in a field setting. The authors are currently working to develop an instrument that will enable the yield-point metric to be acquired in agricultural field settings.

### Stalk physiology and rind puncture

The data collected in this study indicate that rind puncture resistance is strongly influenced by rind thickness (Fig. 7a). This finding agrees with other reports in the literature [3, 24]. A previous study on recurrent selection for rind penetration resistance that utilized a flat tipped probe and the maximum load metric, produced maize varieties that differed in both rind thickness and diameter [24]. In particular, the high rind puncture population exhibited a very thick rind and small diameters while the low rind puncture population exhibited relatively thin rinds and larger diameters. This is particularly interesting because several researchers have found a *positive* correlation between, diameter and rind puncture resistance (Fig. 7b, c). However, Masole [22] found that even though diameter and rind puncture resistance are positively correlated recurrent selection for rind puncture resistance does not produce stalks with large diameters. In fact, it appears that diameters decrease under selection pressure for rind puncture resistance. In other words, rind penetration is positively correlated with stalk diameter but this does not necessarily imply that breeding for increased rind penetration resistance will increase stalk diameters. This is significant because bending strength and therefore lodging resistance is highly dependent upon the diameter of the stalk, with larger diameter stalks being stronger and more resistant to stalk lodging [10].

### Limitations and future research

Biological tissues possess viscoelastic material properties [25]. As such, the insertion rate utilized during rind penetration experiments is expected to affect the resulting measurement. In addition, different probe geometries may demonstrate different sensitivities to insertion rate. The greatest limitation of this study was the decision (due to practical constraints on sample size, etc.) to hold testing rate constant. Further research will be needed to determine the optimal testing rate for rind puncture of maize stalks. This topic is relevant since high rates are expected to be preferred for high-throughput testing.

Two probes of differing diameters were utilized in this study. The chamfered probe was 2 mm in diameter whereas the pointed probe was 1.5 mm in diameter. The size of the probes was chosen to simplify manufacturing the probes (e.g., smaller diameter probes are increasingly difficult to accurately machine). In addition, we found that probes smaller than those selected in this study could bend or break during testing. The data from this study demonstrates that the shape and geometry of the probe influence the correlation between stalk strength and rind penetration resistance. They likewise indicate that the chamfered probe geometry is superior to the pointed geometry. However, with further investigations a more optimal probe geometry than the chamfered geometry may be discovered.

This study used correlation analyses extensively. Correlation does not imply causality. For example, there is evidence to suggest that the relationship between diameter and rind penetration resistance is not causal as recurrent selection for rind penetration produced stalks with smaller diameters. However, this study and others have shown a positive correlation between diameter and rind penetration resistance. Furthermore, correlation values should be interpreted cautiously since many of the geometric and rind puncture features may covary with one other. Thus, it is not yet entirely clear which of these features has the greatest causal effect on rind penetration metrics. Computational modeling could provide further insights in this area.

## Conclusions

There exists a need in the agricultural and plant science communities to create agreed-upon operating procedures and testing standards related to mechanical traits of plant stems [26]. In particular, a standardized probe geometry and insertion rate for rind penetration studies are needed to enable greater interoperability and meta-analysis of results. Probe geometry has a significant effect on the correlation between rind puncture metrics and stalk bending strength. Of the geometries and rind penetration metrics investigated the chamfered probe and structural yield point metric had the highest correlations with stalk bending strength.

Rind puncture metrics were most closely associated with rind thickness but were also positively correlated with section modulus and stalk diameters. Once again, the chamfered probe exhibited higher correlations with stalk morphology than the pointed probe. Contrary to expectations, rind puncture metrics were not closely related to CT scan intensity or the Young’s Modulus of the rind.

More research will be needed to fully elucidate the relationships between rind puncture, stalk bending strength, stalk lodging and geometric features of the stalk. In particular, further research is needed on the influence of puncture rate, which was not investigated in this study. In the meantime, studies that utilize rind puncture metrics should report detailed information on the probe geometry, puncture rate, and the type of penetration metric used.

## Supplementary information

**Additional file 1.** Text file providing an in depth explanation of the data contained in Additional Files 2 and 3.

**Additional file 2.** Data table in .csv format containing geometric, material property, bending strength, and rind puncture data obtained in this study.

**Additional file 3.** Matlab data table in .mat format containing geometric, material property, bending strength, rind puncture and CT data obtained in this study.

## Data Availability

The data sets obtained and analyzed during the current study are available from the corresponding author upon reasonable request (Additional files 1, 2 and 3).
